# Challenges in Diagnosis and Management of Recurrent Uterine Arteriovenous Malformation: A Case Report

**DOI:** 10.7759/cureus.59665

**Published:** 2024-05-05

**Authors:** Nibedita Maharana, Manas R Behera, Suvradeep Mitra, Sweta Singh

**Affiliations:** 1 Obstetrics and Gynecology, All India Institute of Medical Sciences, Bhubaneswar, Bhubaneswar, IND; 2 Histopathology, Postgraduate Institute of Medical Education and Research, Chandigarh, Chandigarh, IND

**Keywords:** color doppler ultrasound, ultrasound (u/s), total abdominal hysterectomy, uterine artery embolization (uae), s: uterine arteriovenous malformation

## Abstract

Uterine arteriovenous malformation (AVM) is a potentially life-threatening condition. The vast majority of cases are acquired. Uterine artery embolization (UAE) is currently the treatment of choice for symptomatic women desiring future childbearing. However, there is no consensus on the number of UAE procedures that can be performed on an individual woman. We report a case of recurrent uterine AVM and discuss the challenges in diagnosis and management.

A 35-year-old multiparous woman presented with heavy menstrual bleeding (HMB). She had been diagnosed with uterine AVM six years ago and had undergone two previous UAE procedures. Her abdominal examination revealed a healthy Pfannensteil scar. Bimanual examination revealed a normal-sized uterus that was firm, mobile, and fornices were free. Her haemoglobin was 10.2 g/dl. Greyscale two-dimensional ultrasound revealed a normal-sized uterus with multiple hypoechoic lesions in the myometrium. Colour Doppler ultrasound showed intense vascularity with multidirectional flow in the myometrium, suggestive of uterine AVM. In view of previous failed UAE procedures, she opted for a hysterectomy. A total abdominal hysterectomy with a bilateral salpingectomy was performed. Blood loss during the procedure was greater than average, and she was transfused with a unit of packed cells. Her post-operative course was uneventful. Histopathology confirmed the diagnosis of a uterine AVM.

To conclude, the UAE is considered the treatment of choice for symptomatic women with uterine AVM desiring future childbearing. In cases of failure of UAE procedures, hysterectomy is therapeutic but may be associated with more than average blood loss.

## Introduction

Uterine arteriovenous malformation (AVM) is a potentially life-threatening and rare condition [1]. While uterine AVMs may be congenital, the vast majority are acquired [2]. The diagnosis is initially determined by a greyscale ultrasound with colour Doppler [1]. Uterine artery embolization (UAE) is currently considered the treatment of choice for symptomatic women desiring future childbearing [1,2]. However, there is no consensus on the number of such UAE procedures that can be performed on an individual woman in the event of a failure of a UAE procedure. We report a case of recurrent uterine AVM with previous failed UAE procedures and discuss the challenges in diagnosis and management.

## Case presentation

A 35-year-old multiparous woman presented with a history of heavy menstrual bleeding (HMB) of six years duration. Her obstetric score was para two, living two, and an abortion. Her obstetric history was significant because she underwent a suction and evacuation procedure for a molar pregnancy eight years ago. This was followed by two caesarean deliveries six years and three years ago. Her menstrual cycles were regular, lasting for five days, with heavy bleeding per vaginum. She changed seven to eight pads per day. There was an associated mild dysmenorrhoea. Her last menstrual period was two months ago. She had been seen by her local gynaecologist for the HMB two months ago and had been started on tablet norethisterone 5 mg twice daily since then.

Her first episode of HMB had started following her first caesarean delivery. She had been diagnosed with uterine AVM six years ago at a tertiary care hospital, and a UAE procedure had been performed. She had temporary symptomatic relief following this procedure. However, she had a recurrence of symptoms following her second caesarean delivery three years ago. A repeat UAE had been performed at the same centre, with minimal relief of symptoms. She presented to us following a recurrence of her symptoms after two failed UAE procedures.

On examination, there was a mild degree of pallor. Her vitals were stable. There was no thyromegaly. Bilateral breasts and cardiovascular and respiratory system examinations were normal. Abdominal examination revealed a healthy Pfannensteil scar. There were no masses palpable. On speculum examination, the cervix and vagina were healthy, with no bleeding through the os. A bimanual examination revealed a normal-sized, firm uterus that was mobile from side to side, and the fornices were free.

**Figure 1:**
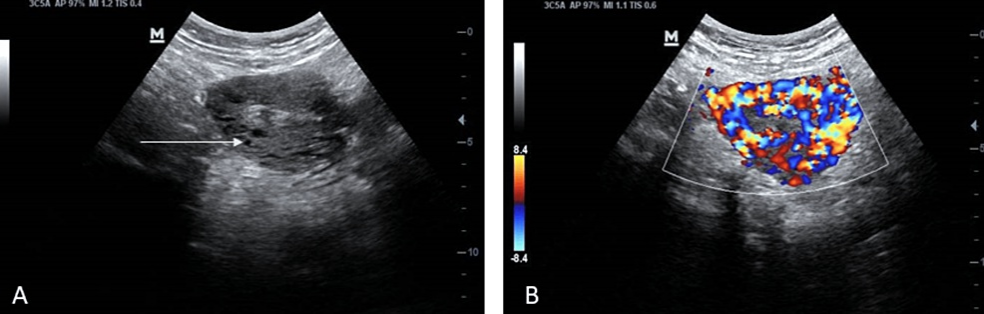
Ultrasound showing (A) Normal-size uterus with multiple hypoechoeic lesions in the myometrium (arrow) on greyscale. (B) Presence of intense vascularity in the myometrium with multidirectional flow, suggestive of uterine arterio-venous malformation (AVM) on colour Doppler.

Her haemoglobin was 10.2 g/dl. Greyscale, two-dimensional transabdominal ultrasound revealed a normal-sized uterus with multiple hypoechoic lesions in the myometrium (Figure 1A). She refused a transvaginal ultrasound.

Colour Doppler ultrasound showed intense vascularity with multidirectional flow in the myometrium (Figure 1B), suggestive of uterine AVM. The patient was advised computed tomography (CT) angiography for better characterization of the lesion but declined as the same had been performed prior to the second sitting of the UAE. She also refused to undergo magnetic resonance imaging (MRI) due to the cost factor. Her serum β human chorionic gonadotrophin (hCG) was within the normal limit.

**Figure 2:**
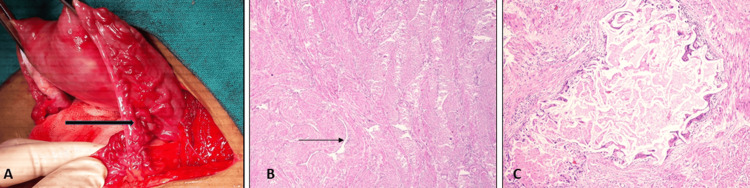
Operative and histopathology imaging showing (A) Multiple tortuous vessels (black arrow) over the right fallopian tube and parametrium. (B) Angulated thick and thin-walled vascular channels (arrow) within the myometrium (hematoxylin and eosin, 40×). (C) A dilated vessel containing extraneous thrombogenic agent with giant cell reaction and perivascular inflammation around it (hematoxylin and eosin, 200×).

She was offered a choice of repeat UAE, medical management with progesterone, or a hysterectomy as a last resort. In view of previous failed UAE procedures, a symptomatic condition compromising a good quality of life (QOL), and having completed childbearing, the patient opted for hysterectomy. A total abdominal hysterectomy with bilateral opportunistic salpingectomy was performed under the combined spinal epidural block. Intraoperatively, the uterus was normal in size with the presence of multiple tortuous vessels over the anterior aspect of the uterus, the utero-vesical fold of the peritoneum, bilateral fallopian tubes, round ligaments, and parametrium (Figure 2A).

There was excessive bleeding during the separation of the utero-vesical fold of the peritoneum before clamping the uterine vessels, probably due to the extensive vasculature. Blood loss during the procedure was around 1000 ml, which was more than average. She was given 1 unit of packed cell transfusion intra-operatively. Her post-operative course was uneventful, and she was discharged on the fourth post-operative day.

She was reviewed at two weeks post-surgery and was doing well. Histopathology reports revealed the presence of angulated thick and thin-walled vascular channels within the myometrium (Figure 2B), confirming the clinical diagnosis of uterine AVM. A few vessels revealed the presence of extraneous thrombogenic agent with perivascular inflammation and giant cell reaction (Figure 2C), confirming the previous UAE procedures that had been correctly performed. On follow-up at six weeks, the patient was asymptomatic and doing well.

Patient perspective

At admission, the patient was worried about her QOL as a mother of two young children. In her words, her menstrual flow was ‘like a tap’, which would start and stop abruptly, with a heavy flow for five days. She was dissatisfied due to the failure of UAE procedures that had been done twice before, which were costly, too. She was delighted with the treatment she received. At the six-week follow-up, she said that it was the longest time she had been without any medications for controlling her heavy menstrual bleeding and was very happy and satisfied.

## Discussion

Uterine AVM is a potentially life-threatening and rare condition [1]. Uterine AVMs may be congenital or acquired and consist of the proliferation of venous and arterial channels with fistula formation and a mixture of capillary-like vessels [2]. Congenital AVMs are less common and result from the abnormal development of primitive vessels, leading to abnormal connections between the arteries and veins in the uterus without an interconnecting capillary bed. Acquired uterine AVMs are more common and may be caused by communications between the uterine arteries and the myometrial veins, secondary to iatrogenic events like caesarean delivery, termination of pregnancy, dilatation and curettage, or pathological conditions like multifetal gestation or miscarriage [3]. In our case, probably her first caesarean delivery was causative, as she was asymptomatic after her first suction and evacuation procedure for molar pregnancy, though the latter is also an associated finding in many cases.

Currently, the initial diagnosis of uterine AVM is by imaging modalities like ultrasound, colour Doppler, and spectral Doppler in a woman presenting with AUB [3-5]. Greyscale ultrasound of uterine AVM commonly shows heterogeneous nonspecific spaces in the myometrium, multiple cystic lesions, and tubular anechoic areas concomitant with a normal endometrium [3]. Colour Doppler ultrasound shows these lesions as hypervascular with turbulent flow and multiple tortuous feeding vessels [4]. Spectral Doppler ultrasound shows low resistance and pulsatility indices with a high peak systolic velocity flow [5]. Digital subtraction angiography is considered to be the gold standard for the diagnosis of uterine AVM but is limited by its invasive nature [3]. Besides magnetic resonance imaging with or without contrast, CT, CT angiography, and hysteroscopy have also been used at various stages of the diagnosis of uterine AVMs [3]. MRI and CT angiography have the added advantage of detailing the uterine AVM and also its relationship with the surrounding structures, which may help in guiding management [6,7].

The differential diagnosis for uterine AVMs is retained products of conception (RPOC), gestational trophoblastic neoplasia (GTN), and vascular pseudoaneurysm [3]. RPOC and GTN may be considered when the serum β hCG is elevated, and colour Doppler ultrasound shows increased vascularity in the region of the endometrium. In cases of uterine artery pseudoaneurysms, colour Doppler ultrasound shows a blood-filled cystic structure with swirling arterial flow in the myometrium, while in cases of AVMs, an intense vascular tangle with high velocity and low-resistance arterial flow is visualized [8].

UAE has emerged as the treatment of choice in recent times, though conservative approaches with progesterone and gonadotropin-releasing hormone agonists have also been used [9]. However, there are no protocols to guide which women should be managed with hormones, when to offer UAE, how many UAE procedures may be offered to an individual woman, or when to switch over to hysterectomy for symptom alleviation. The International Federation of Gynaecology and Obstetrics lists AVM under the “not yet classified” subgroup as a cause of abnormal uterine bleeding (AUB) and says that these entities have been poorly defined, inadequately examined, or both [10]. Historically, cases of uterine AVM were diagnosed at laparotomy or hysterectomy.

Failure of embolization procedures may be due to the type of embolic material used, the expertise of the intervention radiologist, or a regrowth of the AVMs. In our case, histopathology demonstrated the presence of embolic material at the correct site. Hence, probably, the regrowth and extensive nature of the AVM were responsible for the failure of the UAE procedure. Hysterectomy for uterine AVM may be associated with more than usual blood loss, as seen in our case, probably due to extensive AVM at sites like the utero-vesical fold of the peritoneum.

## Conclusions

In a woman of reproductive age presenting with AUB, ultrasound aids in establishing the initial diagnosis of uterine AVM. Greyscale ultrasound may show heterogeneous nonspecific spaces, multiple cystic lesions, and tubular anechoic areas in the myometrium, concomitant with normal endometrium. CD ultrasound may show these lesions as hypervascular with turbulent flow and multiple tortuous feeding vessels. The UAE is considered the treatment of choice for symptomatic women desiring future childbearing. However, there is no consensus on the number of UAE procedures that may be offered to an individual woman. In cases of failed UAE procedures, hysterectomy is therapeutic, improving the patient’s QOL, but may be associated with more than average blood loss. This case is being reported as the incidence of uterine AVMs is expected to rise, and all obstetricians and gynaecologists should be familiar with its diagnosis and treatment.
